# Maternal Supplementation with Polyphenols and Omega-3 Fatty Acids during Pregnancy: Prenatal Effects on Growth and Metabolism

**DOI:** 10.3390/ani11061699

**Published:** 2021-06-07

**Authors:** Ana Heras-Molina, José Luis Pesántez-Pacheco, Consolación Garcia-Contreras, Marta Vázquez-Gómez, Adrián López, Rita Benítez, Yolanda Núñez, Susana Astiz, Cristina Óvilo, Beatriz Isabel, Antonio González-Bulnes

**Affiliations:** 1 SGIT-INIA, Ctra. De La Coruña Km. 7.5, 28040 Madrid, Spain; delasheras.ana@inia.es (A.H.-M.); jose.pesantez@ucuenca.edu.ec (J.L.P.-P.); congarcon@gmail.com (C.G.-C.); adrian.lopez@inia.es (A.L.); rmbenitez@inia.es (R.B.); nunez.yolanda@inia.es (Y.N.); astiz.susana@inia.es (S.A.); ovilo@inia.es (C.Ó.); 2 Faculty of Agricultural Sciences, School of Veterinary Medicine and Zootechnics, University of Cuenca, Avda. Doce de Octubre, Cuenca 010220, Ecuador; 3 Faculty of Veterinary Medicine, UCM, Ciudad Universitaria s/n, 28040 Madrid, Spain; martavazgomez@gmail.com (M.V.-G.); bisabelr@ucm.es (B.I.); 4 Facultat de Veterinària, Universitat Autònoma de Barcelona, Edifici V, Trav. dels Turons, 08193 Bellaterra, Spain; 5 Departamento de Producción y Sanidad Animal, Facultad de Veterinaria, Universidad Cardenal Herrera-CEU, CEU Universities, C/Tirant lo Blanc, 7, Alfara del Patriarca, 46115 Valencia, Spain

**Keywords:** antioxidants, fatty-acids, intrauterine-growth-restriction, pregnancy, swine-model

## Abstract

**Simple Summary:**

The present study aimed to determine benefits and risks of a dietary supplementation combining hydroxytyrosol and n-3 polyunsaturated fatty acids (PUFA) on prenatal development and metabolic traits in swine, a model of intrauterine growth restricted (IUGR) pregnancies. No effects were found regarding sows’ weight and adiposity. Treated sows had larger litters, with smaller fetuses. However, these animals had better development of some major organs. Fetuses from the treated group had better glycemic and lipidic indexes, but no effects on anti/prooxidant profiles were found.

**Abstract:**

Maternal supplementation with antioxidants and n-3 PUFAs may be a promising strategy to reduce the risk of intrauterine growth restriction and preterm delivery, which may diminish the appearance of low-birth-neonates. A previous studies showed beneficial outcomes of the combination of hydroxytyrosol and linoleic acid, but there is no data of its prenatal effects. The present study aimed to determine the possible prenatal implications of such maternal supplementation at prenatal stages in swine, a model of IUGR pregnancies. Results showed effects on litter size, with treated sows having larger litters and, therefore, smaller fetuses. However, the brain/head weight ratio showed a positive effect of the treatment in development, as well as in some other major organs like lungs, spleen, or kidneys. On the other hand, treated piglets showed better glycemic and lipidemic profiles, which could explain postnatal effects. However, further research on the implications of the treatment on litter size and prenatal and postnatal development must be done before practical recommendation can be given.

## 1. Introduction

Maternal nutrition during pregnancy is critical for fetal growth, with major implications in the developmental competence and health status of the offspring during its lifetime [1]. Thus, in pigs, having in mind their importance for pork production and as a biomedical model, different nutrients for mothers are being tested to improve the fetal status. Maternal supplementation with polyphenols during pregnancy is being thoughtfully studied because of their antioxidant properties [2] and, more specifically, the interest in using hydroxytyrosol (a polyphenol present in olive oil and fruits) is increasing not only due to its antioxidant capacity, but also because of its metabolism-regulatory, anti-inflammatory, and immuno-modulatory properties [3]. Hydroxytyrosol has also been shown to diminish lipid peroxidation in swine fetuses, increasing their availability of omega-3 and omega-6 polyunsaturated fatty acids (n-3 and n-6 PUFA) [4]. Our group has previously shown the usefulness of this compound to counteract the appearance of intrauterine growth restriction (IUGR) and, therefore, to diminish the incidence of low birth weight (LBW) neonates and to favor the postnatal development of the piglets [4,5,6,7].

The positive role of hydroxytyrosol on fetal availability of n-3 and n-6 PUFA is pivotal, since PUFAs are molecules with key functions in metabolism, cell structure, and signaling being vital for the adequate fetal development during pregnancy (as reviewed in [8]). Notably, n-3 and n-6 PUFAs are indispensable for adequate tissue development during fetal stages and, therefore, pregnancy success [9]. Such importance is boosted by the fact that these fatty acids are deemed essential due to the inability of the animals to synthesize them. Thus, the fetus must obtain these essential fatty acids (EFA) through maternal transfer [10].

Having in mind the importance of the dietary intake of EFA by the mother on fetal development and homeostasis, maternal supplementation with α-linolenic acid (ALA, which is a precursor of other PUFAs), or directly with EPA and DHA [11], have increased in popularity [12]. However, there is evidence that excessive PUFAs intake may be detrimental for the fetal and newborn health status [13,14]. Therefore, there is a necessity of a systematic risk–benefit analysis and interventional research on PUFA supplementation during pregnancy, to further understand their effects at prenatal and postnatal stages in IUGR pregnancies, as recommended by the World Health Organization (WHO) (https://www.who.int/elena/titles/fish_oil_pregnancy/en/ (accessed on 17 January 2021)).

A main concerning issue in the use of PUFA supplementation during pregnancy is the negative effects on the oxidative/antioxidant status and homeostasis [15,16,17]. To counteract this oxidative effect of PUFAs, an antioxidant is needed. In this regard, our group has studied the combination of n-3 PUFA with hydroxytyrosol in the maternal diet [18]. The results obtained indicated that the offspring from supplemented sows had a lower mean weight and corpulence at birth, but a higher growth rate and, thus, higher weight and corpulence, increased muscle development with similar adiposity and better lipidemic profile at juvenile stages compared with mothers given a diet with no hydroxytyrosol and n-3 PUFA supplementation. Despite the promising results obtained, there is a scarcity of both the realistic benefits and the potential hazards of supplementation with PUFAs and hydroxytyrosol during pregnancy. Thus, the present trial aimed to determine the effects of a maternal dietary supplementation combining hydroxytyrosol and n-3 PUFAs on developmental patterns and metabolic traits of fetuses at IUGR risk at prenatal stages.

## 2. Materials and Methods

### 2.1. Ethic Statement

The experiment was performed according to the Spanish Policy for Animal Protection (RD 53/2013), which meets the European Union Directive 2010/63/UE on the protection of research animals. The INIA Committee of Ethics in Animal Research assessed and approved the experimental procedures (report CEEA 2013/036, 19 February 2014). Sows were housed in INIA animal facilities, which are in accordance with local, national, and European requirements for Scientific Procedures Establishments.

### 2.2. Animals and Experimental Procedures

The study involved a total of 131 fetuses (60 from the control group and 71 from the treated group), obtained from 14 primiparous Iberian sows pregnant after cycle synchronization with altrenogest (Regumate^®^, MSD, Boxmeer, The Netherlands) and artificial insemination with cooled semen from the same purebred boars.

Fetuses were obtained on gestational day 100 (which corresponds approximately with 90% of the 112-days gestation typical of this breed). On this day, blood samples were drawn from the orbital sinus of the sows, after 16 h fastening with sterile EDTA 10 mL vacuum tubes (Vacutainer™ Systems Europe, Meylan, France). Samples were immediately centrifuged at 1500× *g* for 15 min and afterwards, the plasma was separated and biobanked into polypropylene vials at −80 °C until they were assayed for metabolic biomarkers (i.e., glycemic values and lipid profiles) and antioxidant/oxidative status.

During pregnancy, sows were fed with a standard grain based-feed diet with the following mean component values: dry matter, 89.9%; crude protein, 12.28%; fat, 3.55%; metabolizable energy, 2910.44 Mcal/kg. From the start of the experimental period (insemination day, Day 0) to gestational day 35, food amount was adjusted to fulfill individual daily maintenance requirements based on data from the British Society of Animal Health [19]. Most abundant fatty acids (FA) in the diet were palmitic acid (20.6%), oleic acid (19.3%), and linolenic acid (41.5%).

On gestational day 35, all sows were weighed, and the feed amount was adjusted to fulfill 50% of daily maintenance requirements until delivery. This diet restriction has been previously found to increase the incidence of intrauterine growth restriction (IUGR; [20]). On this same day 35 of pregnancy, the females were pair-matched by body weight to obtain two homogeneous groups of seven sows. Therefore, there were no differences in mean body weight between groups (149.64 ± 7.47 kg vs. 146.57 ± 2.51 kg; *p* = 0.80), and maternal adiposity in terms of fat amount measured by P2 point (located at 4 cm from the midline and transversal to the head of the last rib) with a multifrequency linear-array ultrasonographic probe (SV1 Wireless scanner, SonopTek, Beijing, China) was also similar (46.67 ± 4.40 vs. 44.57 ± 2.80 cm; *p* = 0.86). One of the groups remained with the same diet (control group, Group C), whereas the other group (treated group, Group T) received an isocaloric diet including 4% of linseed oil and 1.5 mg hydroxytyrosol per kg of feed (Table 1; Natac S.L., Alcorcon, Madrid, Spain). The component values of the diet in the treated group were: dry matter: 89.7%; crude protein: 12.35%; fat: 6.23%; and metabolizable energy, 2.9 Mcal/kg. In the treatment diet, the most prominent fatty acids were palmitic acid (11.82%), linoleic acid (32.26%), and α linoleic acid (28.97%). There were traces of long-chain PUFA in the experimental diets which were probably caused by contamination during feed manufacturing or storage. Fatty acids methyl esters in the diet were identified by a gas chromatograph (Hewlett Packard HP-6890, Santa Clara, CA, USA) with a flame ionization detector and a capillary column (HP-Innowax, 30 m × 0.32 mm i.d. and 0.25 µm polyethylene glycol-film thickness;) [21]; after extraction and methylation by the one-step procedure proposed by Sukhija and Palmquist [22].

### 2.3. Sampling of Fetuses and Placentas

Sows were euthanized in compliance with RD 53/2013. Afterwards, the entire genital tracts were collected for morphometric evaluation and fetal sampling. The content of the uterus was exposed, and fetal sex was determined by visual inspection immediately after recovery. A sample of fetal blood was drawn from the heart and/or umbilical cord using EDTA syringes and processed as previously described for sows.

Head size (biparietal diameter and occipito–nasal length), trunk length (crown–rump length) and corpulence (thoracic and abdominal circumferences) were measured in all individuals. Then, the head was separated from the trunk at the atlanto–occipital union and weighted. Total viscera were extracted from the body and carcass and total viscera were weighted separately. Main organs (brain, heart, lungs, liver, kidneys, intestine, and spleen) were also weighted individually and, finally, ratios of head-to-body weight, brain-to-head weight, and weight of total viscera and individual organs relative to viscera weight were calculated [5,23].

### 2.4. Evaluation of Maternal and Fetal Anti/Prooxidant and Metabolic Status

Values for total antioxidant capacity were determined using the ferric reducing antioxidant power assay (FRAP) as previously described [24], whilst lipid peroxidation was assessed by measuring malondialdehyde (MDA; µmol/L) using the thiobarbituric acid reaction [25].

Parameters related to glycemic profile (glucose and fructosamine) and lipid metabolism (total cholesterol, high and low-density lipoprotein cholesterol [HDL-c and LDL-c, respectively] and triglycerides), lactate and urea were measured in maternal and fetal plasma using a clinical chemistry analyzer (Konelab 20, Thermo Scientific, Vantaa, Finland), according to manufacturer’s instructions [26].

### 2.5. Statistical Analysis

Data were analyzed using SPSS 25.0 (IBM Corp., Armonk, NY, USA). Data from sows were analyzed via Student’s *t*-test and a general linear model (GLM) for repeated measures (body weight and subcutaneous adiposity). Verification of normal distribution was done with a Shapiro–Wilk test. The equality of variance was studied with a F-test. Effects of diet (control vs. treatment) sex (female vs. male) on developmental traits, adiposity, fatty acid composition, oxidative stress, and metabolic status were assessed using two-way ANOVA and Student’s *t*-test. Due to the strong bias between treatment and litter size, litter size was also considered an effect. To group animals by their litter size, we calculated the total mean number of fetuses per litter (9.25 ± 1.42 piglets per sow), and then defined small litter as those with ≤9 piglets and large litters as those with >9 piglets. Chi-squared and Fisher tests were used to ascertain differences in the proportions of small/large litters, females/males, and IUGR e between treatments.

Relationships between maternal metabolic biomarkers and features of fetuses were explored using Pearson correlation. Based on previous studies [27], fetuses with severe growth restriction (IUGR) were defined as those having a body weight lower than one standard deviation of the litter mean value. Statistical significance was considered when *p* < 0.05, whereas a trend was considered when 0.1 < *p* < 0.05.

## 3. Results

### 3.1. Effects of Dietary Hydroxytyrosol and n-3 PUFA on the Sows

There were no main differences of the treatment on the morphometrics and metabolic features of the sows. At the day of sampling (Day 100 of pregnancy), body weight and adiposity (in terms of total subcutaneous fat and outer and inner layers apart) and plasma indexes for pro-/antioxidant status and metabolism of glucose and lipids were similar in both control sows (Group C) and sows treated with hydroxytyrosol and n-3-PUFA (Group T), as depicted in Supplementary Table S1.

### 3.2. Effects of Dietary Hydroxytyrosol and n-3 PUFA on the Fetuses

#### 3.2.1. Effects on Litter Features

There was a trend (*p* = 0.075), for a higher mean litter size in the Group T (9.86 ± 0.55 vs. 8.42 ± 0.76 piglets/sow in the Group C), and there was a higher proportion of large litters in the Group T compared with Group C (19 vs. 77%, *p* < 0.00001). However, no significant differences were found in the number of ovulations (12.33 ± 1.0 in Group C vs. 12.71 ± 0.86 in the Group T; *p* = 0.78) or the ratio of viable fetuses compared to the number of ovulations (71.48 ± 0.03 vs. 79.16 ± 0.06% in Groups C and T, respectively; *p* = 0.33). Finally, the percentage of females and males were 47% and 52%, respectively, in Group C, whereas there was 58% of females and 42% of males in the treated group, which means a tendency of different proportions of females and males in the Group T (*p* = 0.08). Further information about litter size and composition per sow can be found at Supplementary Table S2.

#### 3.2.2. Effects on Body Weight, Size, and Composition

**Figure 1:**
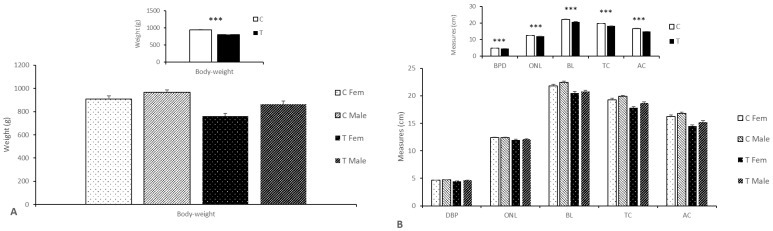
Mean (±S.E.M.) body weights (panel **A**) and sizes (panel **B**) ± S.E.M. of fetuses born from control sows (Group C) or sows treated with hydroxytyrosol and n-3 PUFA from Day 35 to Day 100 of gestation (Group T). Main graphs represent sex-and group-differences, while insets represent only group-related differences. C = control; T = treated; BPD = biparietal diameter; ONL = occipito–nasal length; TL = trunk length; TC = thoracic circumference; AC = abdominal circumference. *** *p* < 0.001.

The uterine weight was higher in the sows from the Group C (16.86 ± 0.70 kg vs. 16.14 ± 1.04 kg; *p* < 0.05) despite of a trend for a higher number of fetuses in the Group T. This outcome was due to the higher weight of the control fetuses and placentas when compared to their treated counterparts (938.1 ± 16. 86 g vs. 803.6 ± 18.26 g; *p* < 0.0001 for fetuses and 654.0 ± 18.58 g vs. 519.7 ± 16.97 g; *p* < 0.01 for placentae), without sex-related differences. Overall, there were no sex-related differences, but, conversely, when each treatment was studied separately, female fetuses from treated mothers were lighter than their brothers; no differences were found in the control groups (Figure 1).

The percentage of fetuses with a body weight lower than 1 SD from mean litter weight (defined as IUGR) was 22.0% (13 of 59; 9 females and 4 males) in the Group C, whilst in the Group T such percentage was 14.5% (10 of 69 fetuses; 8 females and 2 males), with no significance (*p* = 0.38). To avoid sex effects (since males are usually bigger than females at this gestational stage and the number of males and females were not equally distributed between groups), we studied each sex separately. When we analyzed only females, there were 17.8% of IUGR in the Group C (5 of 28 female fetuses) and 17.5% in the Group T (7 of 40 female fetuses). When only males were analyzed, Group C had 5 IUGR fetuses out of 31 males (16.1%) and Group T showed 5 IUGR fetuses out of 29 males (17.2%). No significance was achieved in either sex.

Assessment of body size showed that control fetuses were larger than treated fetuses (Figure 1 and Supplementary Table S3), with a longer and wider trunk (*p* < 0.0001), without sex-related differences.

These differences were also driven by litter size. Thus, fetuses from small litters were heavier, longer, and wider than those from large ones (*p* < 0.05 for body weight, trunk length, and abdominal circumference and *p* < 0.1 for occipito–nasal length). However, even in litters with similar size, fetuses in the Group C were bigger than fetuses from Group T (*p* < 0.05).

Similarly to results observed in the entire body weight, the assessment of the weights of carcasses and total viscera were higher in the control group (525.3 ± 10.14 g vs. 442.3 ± 10.8 g; *p* < 0.01 for carcasses and 165.8 ± 3.814 g vs. 138.8 ± 3.458 g; *p* < 0.01 for total viscera; Table 2 and Supplementary Table S3). Sex related effects were only found in the Group T, with females having lighter carcass and viscera than males of the same group (*p* < 0.001 for both). On the other hand, it should be highlighted that carcasses were heavier in the Group C compared to Group T when assessing small litters (*p* < 0.0001), but heavier in the Group T compared to Group C when assessing large litters (*p* < 0.05).

Fetuses from mothers of Group C had heavier weights of the heads and major organs (*p* < 0.05, for all and *p* < 0.1; for brain; Table 2 and Supplementary Table S3). Independently of treatment group, fetuses from small litters had a heavier head, spleen, and intestine than fetuses from large ones (*p* < 0.05).

The assessment of the different ratios between main organs and structures (Table 2 and Supplementary Table S4) showed that fetuses in the Group T showed higher brain/head weight ratio (*p* < 0.0001); carcass/total weight ratio (*p* < 0.01); total viscera weight/total weight ratio (*p* < 0.05). Such effect was also found in the ratios of spleen and kidneys to total viscera weight (*p* < 0.05, for both). There were significant interactions with litter size in brain/head ratio, liver/total viscera weight and intestines/total viscera weight, being higher in small litters in the case of Group C and in large litters in Group T (*p* < 0.05 for all).

#### 3.2.3. Effects on Fetal Pro-Antioxidant and Metabolic Status

There were no significant differences between groups when either antioxidant capacity (in terms of ferric reducing antioxidant power assay; FRAP) or lipid peroxidation (in terms of malondialdehyde; MDA) were analyzed at plasma samples. We observed higher MDA levels in females (Group C: 2.2 ± 0.1 vs. 2.0 ± 0.1 and Group T: 2.0 ± 0.1 vs. 1.7 ± 0.1; *p* < 0.05 for both) independently of treatment and in small litters compared to large ones (2.1 ± 0.1 vs. 1.8 ± 0.1; *p* < 0.05), independently of treatment and sex.

**Figure 2:**
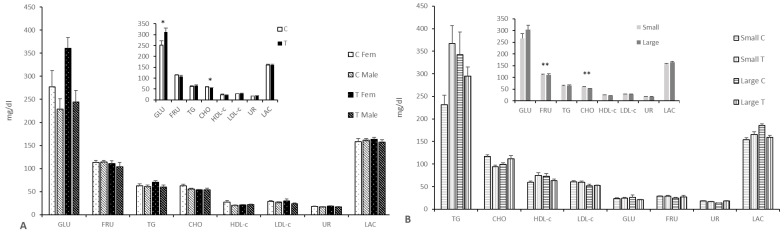
Mean (±S.E.M.) plasma concentrations of indexes for glucids (**A**) and lipids metabolism (**B**) at Day 100 of pregnancy in fetuses from control sows (Group C) and sows treated with hydroxytyrosol and n-3-PUFA from Day 35 to Day 100 of gestation (Group T). Main graphs represent sex-and group-differences, while insets represent only group-related differences. TG = triglycerides; CHO = cholesterol; HDL-c = high density lipoprotein cholesterol; LDL = low density lipoprotein cholesterol; GLU = Glucose; FRUC = fructosamine; UR = urea; LAC = lactate. Asterisks denote significant differences (* for *p* < 0.05 and ** for *p* < 0.01).

The assessment of the glycemic profile showed higher plasma glucose concentrations in the Group T (*p* < 0.05; Figure 2 and Supplementary Table S5) and higher glucose levels in females than in their male counterparts in both groups (*p* < 0.01 for all). Conversely, the assessment of the lipids’ profile showed lower cholesterol levels (*p* < 0.05) in the fetuses of the Group T, especially when comparing females and large litters from both treatment groups (*p* < 0.01 for both). There were significant litter-size by treatment interactions (*p* < 0.05 for fructosamine, glucose, triglycerides, and lactate; *p* < 0.0001 for urea). Thus, glucose, HDL-c, lactate, and triglyceride concentrations were higher in small litters of Group T and large litters of Group C when compared to small litters of Group C and large litters of Group T, respectively. On the other hand, fructosamine and urea were higher in small litters of Group C and large litters of Group T when compared with small litters of Group T and large litters of Group C, respectively.

The assessment of possible feto-maternal interactions in the metabolic profile of the Group C showed no significant relationship for glycemic and lipid profiles. The lactate correlation in this group showed a negative value (r = −0.264; *p* < 0.05), with higher values in the fetuses. Similarly, no significant correlations were found in the glycemic or lipidic profiles in the Group T, with only negative relationships assessed in lactate and urea (r = −0.262, *p* < 0.05 for lactate and r = −0.510, *p* < 0.01 for urea). No significant correlations were found in the MDA and FRAP levels of Group C and T.

## 4. Discussion

In the present study, we aimed to determine the effects of a maternal supplementation with n-3 PUFA and hydroxytyrosol, from day 35 of gestation onwards, on body composition and metabolic and oxidative stress status in sows and their progeny using an animal model that has previously shown to increase the appearance of IUGR offspring [4,5,6,7].

The maternal features such as body weight, oxidative stress, or adiposity did not differ between control and treatment groups, which agrees with previous data in the same swine model of IUGR when only supplemented with dietary hydroxytyrosol [5] or in other animal models in which high dietary levels of n-3 PUFA were given to the dams [27].

The number of fetuses showed a higher litter size due to a significantly higher proportion of large litters in the treated group and a higher ratio of viable fetuses to ovulations, which may reflect a higher offspring viability. In a previous study from our group, hydroxytyrosol did not showed any effect on litter size [6]. On the other hand, Webel et al. [28] described a larger litter size in sows after n-3 PUFA treatment, whilst other studies did not report any difference [29]. Such a higher litter size in Group T plus a trend for a higher proportion of females than males in this group, with lower body weight than their male littermates (a difference not found in the Group C), may have influenced the lower body weight and size found in our treated group compared to controls.

Prolificacy is a well-known factor limiting body weight and size since the uterine space is limited, especially in the Iberian pig [30], because each piglet and its correspondent placenta have less space to develop, and, therefore, reach a smaller size and weight [31]. This outcome has been previously described in pigs treated with hydroxytyrosol but there were no deleterious outcomes at birth and during postnatal phases [4,6] and also in our previous study combining n-3 PUFA and hydroxytyrosol [18].

The results on maternal supplementation with n-3 PUFA in previous studies are contradictory, with some of them showing numerically higher body weight at birth [32,33] and other studies showing no effect [34]. These controversial results have been also reported in humans (reviewed by Grieger and Clifton, [35]). These incongruences could be explained by the different source and concentration of n-3 PUFA supplemented, as well as plausible implications of lifestyle, so further research on this regard is needed before any recommendation.

Similarly to the higher birth-weight, the weights of viscera were heavier in the fetuses from Group C than those from Group T. However, the ratios of brain to head, carcass to body weight, and total viscera to body weight ratio (caused by a higher relative weight of intestine and spleen) were higher in the treated fetuses. The effects on the brain, at the light of the review performed by Innis ([36]) may be related to the positive impacts of n-3 PUFA on brain development and to the neuroprotective properties of hydroxytyrosol [37,38,39]. These differences in the relative weight of brain and intestine may explain the higher average daily weight (ADWG) and growth rate found in a previous study in Iberian pigs with the same maternal supplementation [18]. Moreover, maternal supplementation had different effects depending on the litter size in our study. Thus, differences between control and treated animals were more evident when comparing small litters than when comparing large ones, in agreement with previous studies [6]. Therefore, these results suggest better results of the hydroxytyrosol and n-3 PUFA supplementation in larger litters.

Differences in developmental patterns between Groups C and T may be related to metabolic features; specifically, glucose (higher in treated fetuses). Glucose is the metabolite that crosses the placenta at easiest [40,41], thus, any improvement in placental circulation increases the glucose availability to the fetus. This is vital on fetal development (specially the brain) [42,43] and neonatal survival, since the newborn pig depends on its glucose energy reserves to move to the mother and suck, and to regulate body temperature [44]. It is also important to note that glucose levels were different between groups depending on litter size: higher in the small litters of the Group T and higher in the large litters of the Group C. Conversely, fructosamine (a molecule indicative of precedent glucose availability [45]) was higher in the small litters of the Group C and in the large litters of the Group T. Thus, the treatment affected the glycolytic profile differently depending on the litter size, resulting in better glucose metabolism in large litters. Assessment of lipid metabolism showed that cholesterol levels were lower in the Group T, in accordance with previous research [46,47]. The interaction between treatment and litter size also affected other metabolites related to lipid metabolism, like HDL-c and triglycerides (higher in small litters in the Group T and in large litters in the Group C when compared with the opposite treatment and the same litter size), whereas the opposite was found in LDL-c. Previous research found that both n-3-PUFA and hydroxytyrosol have important effects on lipid metabolism, with n-3 PUFA reducing plasma concentrations of total cholesterol and LDL-c [48], as well as controlling plasma triglyceride levels at postprandial states [49]. On the other hand, hydroxytyrosol has been demonstrated to increase plasma levels of HDL-c while decreasing total cholesterol and LDL-c both in humans [50] and pigs [6]. However, it is important to note that, in the present trial, the correlation in metabolic parameters between mothers and piglets is low. One of the limitations of the study is the relatively low number of sows used, so further research is needed to fully understand the processes implicated in differences between Group C and Group T fetuses. Another possible explanation is the capability of the fetus to achieve a certain level of metabolic autonomy, especially in situations of maternal nutritional changes and challenges (as reviewed by [51]).

## 5. Conclusions

The present study shows that maternal supplementation with hydroxytyrosol and n-3 PUFA has differential effects on the growth and metabolism of the offspring at prenatal stages depending on the litter size. Overall, fetuses from small litters were heavier and bigger, with different plasma metabolites than those from large litters. However, Groups C and T behaved differently in small and large litters, especially at the brain level, but also at the glucose metabolism, probably preventing newborn piglets from death due to the higher brain development during fetal stages, as well as for better thermoregulation. Nevertheless, even with the positive outcomes in the percentage of IUGR and metabolites, more research is needed previously on mechanistic issues and prenatal and postnatal outcomes previously to any advice of use in pregnant females. Also, more studies would be needed to ascertain the beneficial results in swine production.

## Figures and Tables

**Table 1:** Estimated analysis (g/kg), ingredient (g/kg; left table), and fatty acid composition (g/100 g total fatty acids) of the experimental diets of Control sows (group C) and treated with hydroxytyrosol and n-3-PUFA (Treated; group T).

							g/100 g Total Fatty Acids	
Estimated Analysis g/kg	Group C	Group T	Ingredient g/kg Dry Matter Basis	Group C	Group T	Fatty Acids	Group C	Group T
Dry Matter (DM)	910.7	910.3	Barley	234.0	299.0	C14:0	0.463	0.198
EM (Kcal/kg)	2910.4	2909.1	Wheat	300.1	192.5	C16:0	20.64	11.83
Crude protein	122.8	123.4	Wheat bran	245.5	300.0	C16:1 n-9	0.203	0.161
Crude fat	35.5	62.3	Cookie Flour	100.0	0.0	C16:1 n-7	0.867	0.192
Crude fiber	62.1	73.0	Beet pulp	50.0	50.0	C17:0	0.469	0.234
			Sunflower flour (28%)	31.5	68.0	C18:0	3.695	3.232
			Soybean oil	7.0	0.0	C18:1 n-9	19.26	18.38
			Linseed oil	0.0	40.0	C18:1 n-7	4.736	3.101
			Sepiolite	0.0	18.1	C18:2 n-6	41.51	32.26
			Salt	5.0	5.0	C18:3 n-3	4.132	28.96
			Phosfate Monocalcium	2.5	2.5	C20:0	0.349	0.288
			Calcium carbonate	16.5	17.5	C20:1 n-9	0.904	0.442
			L-Lysine (500 g/kg)	4.2	3.8	C20:5 n-3	0.234	0.105
			L-Threonine	0.7	0.6	C22:4 n-6	0.122	0.093
			MVP	3.0	3.0	C22:5 n-3	1.248	0.287
						C22:6 n-3	1.151	0.225
						SFA	25.62	15.78
						MUFA	25.97	22.28
						PUFA	48.39	61.93
						n-6	41.63	32.35
						n-3	6.766	29.58
						n-6/n-3	6.153	1.094

MVP = mineral and vitamin premix; SFA = sum of saturated fatty acids; MUFA = sum of monounsaturated fatty acids; PUFA = sum of polyunsaturated fatty acids; n-6 = sum of omega-6 PUFA; n-3 = sum of omega-3 PUFA.

**Table 2:** Mean (± S.E.M.) values of weight of different body parts and major organs and their ratios at Day 100 of gestation in different treatments (fetuses from control sows, Group C and from sows treated with hydroxytyrosol, and n-3-PUFA from Day 35 to Day 100 of gestation (Group T) and by litter sizes (small ≤ 9 fetuses, large > 9 fetuses).

		Treatment		Litter Size	
		Control	Treated	Small	Large
Weights (g)					
Head		207.4 ^E^ ± 2.94	183.4 ^F^ ± 3.17	200.5 ^A^ ± 3.33	188.4 ^B^ ± 3.37
Carcass		525.3 ^E^ ± 10.1	442.3 ^F^ ± 10.8	500.8 ^A^ ± 11.4	460.2 ^B^ ± 11.6
Viscera		165.8 ^E^ ± 3.81	138.8 ^F^ ± 3.46	156.7 ± 4.16	145.7 ± 3.71
Brain		29.9 ± 0.23	29.3 ± 0.23	29.5 ± 0.19	29.7 ± 0.28
Heart		9.09 ^E^ ± 0.24	7.70 ^F^ ± 0.19	8.63 ± 0.25	8.0 ± 0.20
Lungs		31.8 ^E^± 0.80	27.9 ^F^ ± 0.76	30.4 ± 0.78	29.0 ± 0.84
Liver		28.4 ^E^ ± 0.81	24.9 ^F^ ± 0.72	27.3 ± 0.82	25.6 ± 0.76
Intestines		54.6 ^E^ ± 1.50	44.5 ^F^ ± 1.17	51.8 ^C^ ± 1.58	46.4 ^D^ ± 1.26
Kidneys		7.90 ^E^ ± 0.19	6.61 ^F^ ± 0.17	7.55 ^C^ ± 0.20	6.84 ^D^ ± 0.18
Spleen		1.98 ^C^ ± 0.05	1.78 ^D^ ± 0.05	1.97 ^A^ ± 0.05	1.78 ^B^ ± 0.05
Ratios					
Head	/Total	0.398 ^C^ ± 0.002	0.231 ^D^ ± 0.002	0.226 ± 0.002	0.228 ± 0.002
Carcass		0.315 ^E^ ± 0.003	0.549 ^F^ ± 0.002	0.559 ^C^ ± 0.002	0.549 ^D^ ± 0.003
Viscera		0.176 ^A^ ± 0.002	0.172 ^B^ ± 0.001	0.174 ± 0.001	0.174 ± 0.001
Brain	/Head	0.146 ^E^ ± 0.002	0.162 ^F^± 0.003	0.149 ^C^ ± 0.002	0.160 ^D^ ± 0.003
Heart	/Viscera	0.055 ± 0.001	0.056 ± 0.001	0.055 ± 0.001	0.056 ± 0.001
Lungs		0.193 ^A^ ± 0.003	0.202 ^B^ ± 0.003	0.196 ± 0.003	0.200 ± 0.003
Liver		0.173 ± 0.003	0.179 ± 0.003	0.175 ± 0.002	0.178 ± 0.004
Intestines		0.330 ^A^ ± 0.004	0.321 ^B^ ± 0.003	0.329 ^A^ ± 0.003	0.320 ^B^ ± 0.003
Kidneys		0.048 ^A^ ± 0.001	0.048 ^B^ ± 0.001	0.048 ± 0.001	0.048 ± 0.001
Spleen		0.012 ^A^ ± 0.000	0.013 ^B^ ± 0.000	0.013 ± 0.000	0.012 ± 0.000

^A,B^: *p <* 0.05; ^C,D^: *p <* 0.01; ^E,F^: *p <* 0.001.

## Data Availability

All data are contained in the article or supplementary material.

## Supplementary Materials

The following are available online at https://www.mdpi.com/article/10.3390/ani11061699/s1, Table S1: Mean (±S.E.M.) of body weight, subcutaneous fat, pro-/antioxidant and metabolic status in control sows (Group C) and treated with hydroxytyrosol and n-3-PUFA from Day 35 to Day 100 of gestation (Group T) at the end of the experiment; Table S2: Detailed information (total number and percentage) on sex (total, male, and female fetuses) and weight distribution (presence of IUGR animal) in the pregnancies studied in the current trial; Table S3: Mean (±S.E.M.) of body measures and weights of fetuses born from control sows (Group C) or sows treated with hydroxytyrosol and n-3 PUFA from Day 35 to Day 100 of gestation (Group T) by treatment and sex (Table A) and by litter size and treatment (Table B); Table S4: Mean (±S.E.M.) of weights ratios of fetuses born from control sows (Group C) or sows treated with hydroxytyrosol and n-3 PUFA from Day 35 to Day 100 of gestation (Group T) by treatment and sex (Table A) and by litter size and treatment (Table B); Table S5: Mean (±S.E.M.) of plasma concentrations of indexes for glucids and lipids metabolism of fetuses born from control sows (Group C) or sows treated with hydroxytyrosol and n-3 PUFA from Day 35 to Day 100 of gestation (Group T) by treatment and sex (Table A) and by litter size and treatment (Table B).
